# Pathophysiological roles of FGF signaling in the heart

**DOI:** 10.3389/fphys.2013.00247

**Published:** 2013-09-06

**Authors:** Nobuyuki Itoh, Hiroya Ohta

**Affiliations:** Department of Genetic Biochemistry, Kyoto University Graduate School of Pharmaceutical SciencesKyoto, Japan

**Keywords:** FGF, heart, hypertrophy, fibrosis, heart failure, cardiomyokine

## Abstract

Cardiac remodeling progresses to heart failure, which represents a major cause of morbidity and mortality. Cardiomyokines, cardiac secreted proteins, may play roles in cardiac remodeling. Fibroblast growth factors (FGFs) are secreted proteins with diverse functions, mainly in development and metabolism. However, some FGFs play pathophysiological roles in cardiac remodeling as cardiomyokines. FGF2 promotes cardiac hypertrophy and fibrosis by activating MAPK signaling through the activation of FGF receptor (FGFR) 1c. In contrast, FGF16 may prevent these by competing with FGF2 for the binding site of FGFR1c. FGF21 prevents cardiac hypertrophy by activating MAPK signaling through the activation of FGFR1c with β-Klotho as a co-receptor. In contrast, FGF23 induces cardiac hypertrophy by activating calcineurin/NFAT signaling without αKlotho. These FGFs play crucial roles in cardiac remodeling via distinct action mechanisms. These findings provide new insights into the pathophysiological roles of FGFs in the heart and may provide potential therapeutic strategies for heart failure.

## Introduction

Heart failure represents a major cause of morbidity and mortality and remains a critical health problem. Cardiac hypertrophy and fibrosis subsequently progress to heart failure. Proteins secreted from the heart with functions crucial for its function have been referred to as cardiomyokines. Cardiomyokines may play roles in cardiac remodeling and heart failure. Identifying the underlying molecular targets and novel protective agents of cardiac remodeling is important for improving preventive and therapeutic strategies. Cardiomyokines may be therapeutic targets and/or agents (Fredj et al., 2005; Doroudgar and Glembotski, 2011).

Fibroblast growth factors (FGFs) are signaling proteins of ~150–300 amino acids with diverse functions, mainly in development and metabolism. The mammalian FGF family comprises twenty-two members. FGFs can be classified to as intracellular FGFs (iFGFs), canonical FGFs, and hormone-like FGFs (hFGFs) by their action mechanisms. Intracellular, canonical, and hFGFs are also referred to as intracrine, paracirne, and endocrine FGFs, respectively. Paracrine and endocrine FGFs are secreted signals, mainly in paracrine and endocrine manners, respectively (Itoh and Ornitz, 2011).

Among these FGFs, FGF2, FGF16, FGF21, and FGF23 have been shown to play pathophysiological roles in the heart. These FGFs play distinct roles in cardiac remodeling via unique action mechanisms. Although their action mechanisms remain unclear, these findings provide new insights into the pathophysiological roles of FGFs in the heart and may provide potential therapeutic strategies for heart failure. In this article, we provide a succinct review of the pathophysiological roles of these FGFs in the heart revealed by recent studies using gene-targeted mouse models.

## The FGF and FGFR families

**Figure 1:**
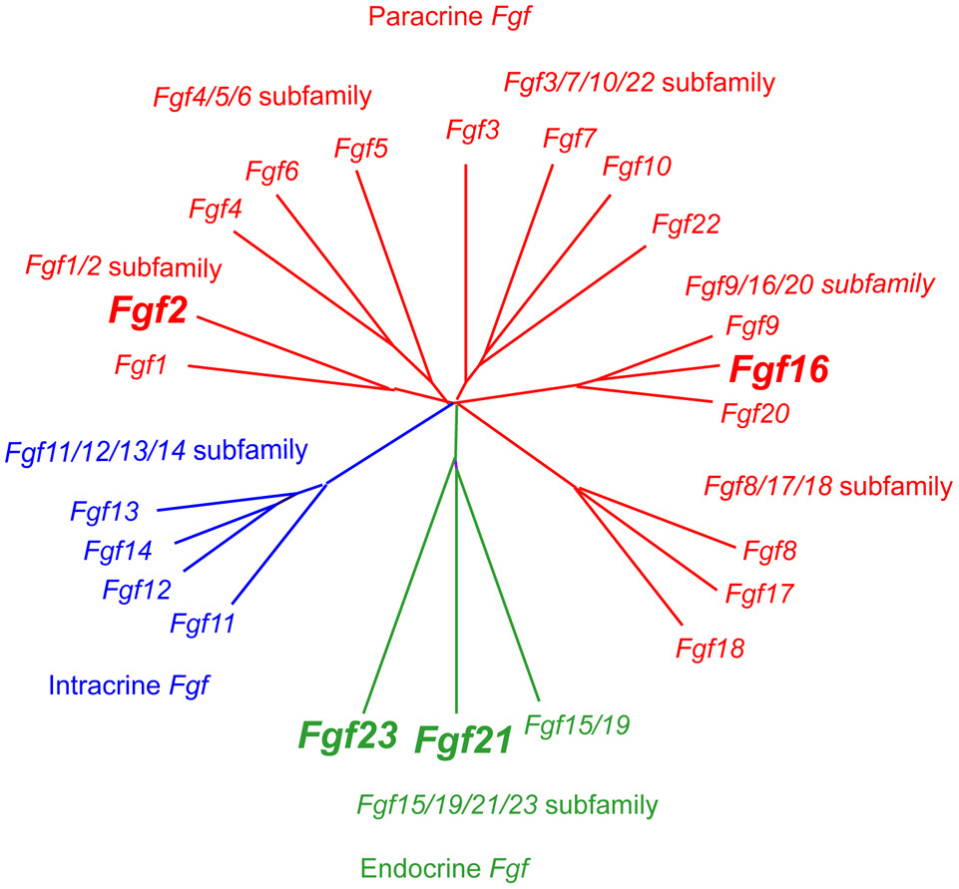
**Evolutionary relationships within the human/mouse *Fgf* gene family by phylogenetic analysis**. Phylogenetic analysis suggests that twenty-two *Fgf* genes can be arranged into seven subfamilies containing two to four members each. Branch lengths are proportional to the evolutionary distance between each gene (Itoh and Ornitz, 2011).

The human/mouse *Fgf* gene family comprises twenty-two members, *Fgf1*-*Fgf23*. *Fgf15*, and *Fgf19* are orthologous genes in mice and humans, respectively. These genes are also referred to as *Fgf15/19*. FGFs are proteins of ~150–300 amino acids with a conserved core of ~120 amino acids with ~30–60% identity. Phylogenetic analysis of the *Fgf* gene family has identified seven subfamilies; *Fgf/1/2, Fgf4/5/6, Fgf3/7/10/22, Fgf8/17/18, Fgf9/16/20, Fgf11/12/13/14*, and *Fgf15/19/21/23* (Figure 1) (Itoh and Ornitz, 2011).

Paracrine FGFs, which comprise fifteen members belonging to the FGF/1/2, FGF4/5/6, FGF3/7/10/22, FGF8/17/18, and FGF9/16/20 subfamilies, mediate biological responses by binding to and activating cell surface tyrosine kinase FGF receptors (FGFRs) with heparin/heparan sulfate as a cofactor. They function as paracrine signaling molecules, of which functions have been mostly studied in development. Endocrine FGFs, FGF15/19, FGF21, and FGF23, also mediate their biological responses in an FGFR-dependent manner. However, they bind to FGFRs with heparin/heparan sulfate with very low affinity. This reduced heparin-binding affinity enables endocrine FGFs to function over long distances in an endocrine manner. They require either αKlotho or βKlotho, which is a single-pass transmembrane protein with a short cytoplasmic domain, as a cofactor instead of heparin/heparan sulfate. They function as hormone-like signaling molecules, mainly in metabolism (Itoh, 2010; Itoh and Ornitz, 2011; Long and Kharitonenkov, 2011; Goetz and Mohammadi, 2013). Intracrine FGFs, FGF11-FGF14, are intracellular proteins that regulate the function of voltage-gated sodium channels in an intracrine manner (Goldfarb, 2012).

Four *Fgfr* genes, *Fgfr1*–*Fgfr4*, have been identified in mammals. These genes encode receptor tyrosine kinases that contain an extracellular ligand-binding domain, transmembrane domain, and split intracellular tyrosine kinase domain. *Fgfr1*–*Fgfr3* encode two major variants generated by alternative splicing. Thus, seven FGFR proteins (FGFRs 1b, 1c, 2b, 2c, 3b, 3c, and 4) with differing ligand-binding specificities have been generated (Zhang et al., 2006).

Paracrine and endocrine FGFs induce the phosphorylation of specific cytoplasmic tyrosine residues in FGFRs. The phosphorylation triggers the activation of cytoplasmic signal transduction pathways, which elicits different cell responses. The activated FGFRs first activate their intracellular substrates, FGFR substrate 2α (FRS2α) and/or phospholipase Cγ1 (PLCγ1). Activated FRS2α initiates downstream signaling through the RAS–MAPK or PI3K–AKT pathway. Although cell responses depend on FGFs and their targeted cells, the RAS–MAPK pathway mainly generates a mitogenic cell response, while the PI3K–AKT pathway mainly promotes cell survival. The activation of PLCγ1 leads to the release of calcium ions from intracellular stores and activation of calcium-dependent signaling. The PLCγ1 pathway is thought to play a role in mediating cell motility (Goetz and Mohammadi, 2013).

## Roles of FGF2 in cardiac remodeling

FGF2 is a paracrine FGF. Although most paracrine FGFs are secreted proteins with N-terminal signal sequences, FGF2 with no signal sequence is not a typical secreted protein. FGF2 may be released from damaged cells or by an exocytotic mechanism that is independent of the endoplasmic reticulum-Golgi pathway (Nickel, 2010). However, FGF2 as well as other paracrine FGFs mediates biological responses as extracellular proteins by binding to and activating FGFRs with heparin/heparan sulfate as a cofactor (Itoh and Ornitz, 2011).

Although *Fgf2* is broadly expressed in mice, *Fgf2* knockout mice are viable. However, cardiac hypertrophy and fibrosis were less developed in *Fgf2* knockout mice with myocardial infarcts (Virag et al., 2007). Isoproterenol-induced cardiac hypertrophy was shown to be protected in *Fgf2* knockout mice (House et al., 2010). The two-kidney one-clip (2K1C) model increased blood renin and angiotensin II levels, leading to a chronic elevation in blood pressure and compensatory cardiac hypertrophy. However, 2K1C-induced cardiac hypertrophy and fibrosis did not develop in *Fgf2* knockout mice (Pellieux et al., 2001).

FGF2 caused hypertrophy by inducing MAPK signaling in cultured cardiomyocytes (Bogoyevitch et al., 1994; Kaye et al., 1996). The cardiac-specific *Fgf2*-overexpression in transgenic mice exacerbated hypertrophy and the effect was blunted in the presence of a pharmacological ERK inhibitor, which also indicated the involvement of FGF2-induced MAPK signaling (House et al., 2010). The heart was shown to predominantly express *Fgfr1c* among *Fgfrs* (Fon Tacer et al., 2010). FGF2 effectively activates FGFR1c with heparin/heparin sulfate in non-cardiac cell types (Zhang et al., 2006). Although these findings do not necessary mean that FGFR1c is a FGF2 receptor in the heart, FGF2 might act on cardiac cells in a paracrine manner and promotes cardiac remodeling by activating MAPK signaling through the activation of FGFR1c (Table 1).

**Table 1:** **Roles of FGFs 2, 16, 21, and 23 in cardiac remodeling**.

	**Remodeling**	**Receptor**	**Signaling**	**Action manner**
FGF2	Promotion	FGFR1c/Heparan sulfate	MAPK	Agonist/Paracrine
FGF16	Prevention	FGFR1c/Heparan sulfate	MAPK	Antagonist/Paracrine
FGF21	Prevention	FGFR1c/βKlotho	MAPK	Agonist/Paracrine
FGF23	Promotion	FGFR1c?	Calcineurin/NEAT	Agonist/Endocrine

## Roles of FGF16 in cardiac remodeling

FGF16 is also a paracrine FGF. *Fgf16* gene is predominantly expressed in the heart (Fon Tacer et al., 2010). However, heart function is essentially normal in *Fgf16* knockout mice (Hotta et al., 2008). Angiotensin II induces cardiac hypertrophy and fibrosis. Transforming growth factor- β_1_ (Tgf- β_1_) acts downstream of angiotensin II and promotes cardiac hypertrophy and fibrosis (Rosenkranz, 2004). Angiotensin II-induced cardiac hypertrophy and fibrosis were shown to be significantly promoted by enhancing *Tgf*- β_1_ expression in *Fgf16* knockout mice (Matsumoto et al., 2013). The response to cardiac remodeling was apparently opposite to that in *Fgf2* knockout mice, as described above.

The biochemical properties of FGF16 are distinct from those of FGF2. *Fgf16* is expressed mainly in cardiomyocytes. FGF16 is efficiently secreted and acts as a paracrine signaling molecule (Hotta et al., 2008). In contrast, FGF2, which is mainly expressed in non-cardiomyocytes, is not a typical secretory protein. FGF2, which is stored in these cells, is released in response to hemodynamic stress (Kaye et al., 1996). FGF2 showed significant proliferative activity in cultured cardiomyocytes, where as FGF16 did not. However, FGF16 antagonized the activity of FGF2 (Lu et al., 2008). FGF16 competed with FGF2 for the binding site of FGFR1c, which is predominantly expressed in the heart (Lu et al., 2008). In addition, FGF2 significantly induced *Tgf*- β_1_ expression in cultured cardiomyocytes and non-cardiomyocytes, where as FGF16 did not. However, FGF16 inhibited FGF2-induced *Tgf*- β_1_ expression, indicating that FGF16 antagonizes FGF2-induced *Tgf*-β_1_ expression. Cardiac *Fgf16* expression was induced after the induction of *Fgf2* expression by angiotensin II. In cultured cardiomyocytes, *Fgf16* expression was promoted by FGF2. These findings indicate a possible mechanism in which FGF16 may prevent cardiac hypertrophy and fibrosis by competing with FGF2 for the binding site of FGFR1c (Table 1) (Matsumoto et al., 2013).

## Roles of FGF21 in cardiac remodeling

FGF21 is an endocrine FGF. FGF21 also mediates its biological responses in an FGFR-dependent manner. However, FGF21 binds to FGFRs with heparin/heparan sulfate with very low affinity. FGF21 efficiently binds to and activates FGFR1c, FGFR2c, and FGFR3c with βKlotho, which is crucial for FGF21 signaling as a cofactor (Kharitonenkov et al., 2008; Suzuki et al., 2008). *Fgf21* is expressed mainly in the liver. *Fgf21* knockout and transgenic mice suggest the diverse metabolic actions of FGF21 in glucose and lipid metabolism (Itoh, 2010; Long and Kharitonenkov, 2011).

*Fgf21* knockout mice also exhibited an increased relative heart weight and develop enhanced signs of dilatation. In response to isoproterenol infusion, cardiac hypertrophy was more enhanced in *Fgf21* knockout mice. FGF21 reversed cardiac hypertrophy in *Fgf21* knockout mice and cultured cardiomyocytes. FGF21 is also produced by cardiomyocytes. Although cardiac FGF21 secretion is lower than hepatic FGF21 secretion, FGF21 is markedly secreted by cardiac cells in response to cardiac stress, and cardiac FGF21 secretion is able to inhibit isoproterenol-induced cardiac hypertrophic damage. Thus, the heart appears to be a target of locally generated FGF21, even though FGF21 is an endocrine FGF. Both *Fgfr1c* and β-*Klotho* are predominantly expressed in cardiomyocytes. These findings indicate that FGF21 acts on cardiomyocytes possibly in a paracrine manner and prevents cardiac hypertrophy by activating MAPK signaling through the activation of FGFR1c with β-Klotho (Table 1) (Planavila et al., 2013).

## Roles of FGF23 in cardiac remodeling

FGF23 is also an endocrine FGF. *Fgf23* knockout mice, which gradually died 4–13 weeks after birth, indicate that FGF23 regulates phosphate and vitamin D metabolism as a bone-derived hormone. FGF23 also mediates its biological responses in an Fgfr-dependent manner. However, FGF23 as well as FGF21 binds to FGFRs with heparin/heparan sulfate with very low affinity. FGF23 was shown to efficiently bind to and activate FGFR1c with αKlotho, which is crucial for FGF23 signaling as a cofactor (Hu et al., 2013).

Chronic kidney disease progression results in elevated serum levels of FGF23, which are associated with left ventricular hypertrophy, a major contributor to cardiovascular disease (Gutiérrez et al., 2009). As α*Klotho* is not expressed in the heart and FGF23 did not activate MAPK signaling in the heart, it has been assumed that FGF23 cannot act directly on the heart to induce injury. However, FGF23 induced hypertrophic growth in cultured cardiomyocytes expressing *Fgfr1c*, but not α*Klotho*. This result suggests αKlotho-independent FGF23 signaling in cardiomyocytes. FGF23 injected directly into the left ventricular myocardium in mice led to the development of left ventricular hypertrophy. FGF23 activated calcineurin/NFAT signaling in cultured cardiomyocytes. These findings indicate that FGF23 promotes cardiac hypertrophy by activating calcineurin/NFAT signaling in an endocrine manner (Table 1) (Faul et al., 2011; Faul, 2012; Touchberry et al., 2013). However, the mechanism of αKlotho-independent FGF23 signaling remains unclear. In contrast, a monoclonal FGF23 neutralizing antibody did not protect rats with chronic kidney disease from cardiovascular injury, indicating that FGF23 might not have direct effects on the heart (Shalhoub et al., 2012).

## Conclusions

FGF2 promotes cardiac remodeling by activating MAPK signaling through the activation of FGFR1c in a paracrine manner. In contrast, FGF16 does not directly act on cardiac cells. FGF16 may prevent cardiac hypertrophy and fibrosis by competing with FGF2 for the binding site of FGFR1c in a paracrine manner. The action mechanism of FGF16 in the heart is unique. In contrast, FGF21 is mainly a liver-derived hormone that regulates glucose and lipid metabolism in an endocrine manner. However, *Fgf21* is also expressed in cardiac cells, although this expression is lower than that in the liver. FGF21 prevents cardiac hypertrophy by activating MAPK signaling through the activation of FGFR1c with β-Klotho. Although FGF21 is essentially an endocrine FGF, FGF21 produced in cardiac cells may act in a paracrine manner. Although both FGF2 and FGF21 activate MAPK signaling in cardiomyocytes, their effects are opposite. The reason for these opposite effects remains unclear. FGF23 is a bone-derived hormone that mainly regulates phosphate and vitamin D metabolism. FGF23 also induces cardiac hypertrophy by activating calcineurin/NFAT signaling. In general, FGF23 signaling is αKlotho/FGFR1c-dependent. However, cardiomyocytes does not express α*Klotho*. It remains unclear what precise FGFR isoform is activated by FGF23 in cardiomyocytes. These FGFs play crucial roles in cardiac remodeling via unique action mechanisms. However, some effects of FGF signaling on cardiac remodeling by these FGFs appear to be contradictory. In addition, it remains unclear which cell types really express *Fgfr1c*, or how cardiomyocytes and non-cardiomyocytes talk to each other by FGF signaling. However, these findings provide new insights into the pathophysiological roles of FGFs in the heart and may provide potential therapeutic strategies for heart failure.

### Conflict of interest statement

The authors declare that the research was conducted in the absence of any commercial or financial relationships that could be construed as a potential conflict of interest.

## References

[B1] Bogoyevitch M. A. Glennon P. E. Andersson M. B. Clerk A. Lazou A. Marshall C. J. (1994). Endothelin-1 and fibroblast growth factors stimulate the mitogen-activated protein kinase signaling cascade in cardiac myocytes. The potential role of the cascade in the integration of two signaling pathways leading to myocyte hypertrophy. J. Biol. Chem. 269, 1110–1119 7507104

[B2] Doroudgar S. Glembotski C. C. (2011). The cardiokine story unfolds: ischemic stress-induced protein secretion in the heart. Trends Mol. Med. 17, 207–214 10.1016/j.molmed.2010.12.003 21277256 PMC3078974

[B3] Faul C. (2012). Fibroblast growth factor 23 and the heart. Curr. Opin. Nephrol. Hypertens. 21, 369–375 10.1097/MNH.0b013e32835422c4 22531163

[B4] Faul C. Amaral A. P. Oskouei B. Hu M. C. Sloan A. Isakova T. (2011). FGF23 induces left ventricular hypertrophy. J. Clin. Invest. 121, 4393–4408 10.1172/JCI46122 21985788 PMC3204831

[B5] Fon Tacer K. Bookout A. L. Ding X. Kurosu H. John G. B. Wang L. (2010). Research resource: comprehensive expression atlas of the fibroblast growth factor system in adult mouse. Mol. Endocrinol. 24, 2050–2064 10.1210/me.2010-0142 20667984 PMC2954642

[B6] Fredj S. Bescond J. Louault C. Potreau D. (2005). Interactions between cardiac cells enhance cardiomyocyte hypertrophy and increase fibroblast proliferation. J. Cell. Physiol. 202, 891–899 10.1002/jcp.20197 15389635

[B7] Goetz R. Mohammadi M. (2013). Exploring mechanisms of FGF signalling through the lens of structural biology. Nat. Rev. Mol. Cell Biol. 14, 166–180 10.1038/nrm3528 23403721 PMC3695728

[B8] Goldfarb M. (2012). Voltage-gated sodium channel-associated proteins and alternative mechanisms of inactivation and block. Cell. Mol. Life Sci. 69, 1067–1076 10.1007/s00018-011-0832-1 21947499 PMC3272111

[B9] Gutiérrez O. M. Januzzi J. L. Isakova T. Laliberte K. Smith K. Collerone G. (2009). Fibroblast growth factor 23 and left ventricular hypertrophy in chronic kidney disease. Circulation 119, 2545–2552 10.1161/CIRCULATIONAHA.108.844506 19414634 PMC2740903

[B10] Hotta Y. Sasaki S. Konishi M. Kinoshita H. Kuwahara K. Nakao K. (2008). Fgf16 is required for cardiomyocyte proliferation in the mouse embryonic heart. Dev. Dyn. 237, 2947–2954 10.1002/dvdy.21726 18816849

[B11] House S. L. House B. E. Glascock B. Kimball T. Nusayr E. Schultz J. E. (2010). Fibroblast growth factor 2 mediates isoproterenol-induced cardiac hypertrophy through activation of the extracellular regulated kinase. Mol. Cell. Pharmacol. 2, 143–154 10.4255/mcpharmacol.10.20 21274419 PMC3026329

[B12] Hu M. C. Shiizaki K. Kuro-o M. Moe O. W. (2013). Fibroblast growth factor 23 and Klotho: physiology and pathophysiology of an endocrine network of mineral metabolism. Annu. Rev. Physiol. 75, 503–533 10.1146/annurev-physiol-030212-183727 23398153 PMC3770142

[B13] Itoh N. (2010). Hormone-like (endocrine) Fgfs: their evolutionary history and roles in development, metabolism, and disease. Cell Tissue Res. 342, 1–11 10.1007/s00441-010-1024-2 20730630 PMC2948652

[B14] Itoh N. Ornitz D. M. (2011). Fibroblast growth factors: from molecular evolution to roles in development, metabolism and disease. J. Biochem. 149, 121–130 10.1093/jb/mvq121 20940169 PMC3106964

[B15] Kaye D. Pimental D. Prasad S. Mäki T. Berger H. J. McNeil P. L. (1996). Role of transiently altered sarcolemmal membrane permeability and basic fibroblast growth factor release in the hypertrophic response of adult rat ventricular myocytes to increased mechanical activity *in vitro*. J. Clin. Invest. 97, 281–291 10.1172/JCI118414 8567946 PMC507016

[B16] Kharitonenkov A. Dunbar J. D. Bina H. A. Bright S. Moyers J. S. Zhang C. (2008). FGF-21/FGF-21 receptor interaction and activation is determined by betaKlotho. J. Cell. Physiol. 215, 1–7 10.1002/jcp.21357 18064602

[B17] Long Y. C. Kharitonenkov A. (2011). Hormone-like fibroblast growth factors and metabolic regulation. Biochim. Biophys. Acta. 1812, 791–795 10.1016/j.bbadis.2011.04.002 21504790

[B18] Lu S. Y. Sontag D. P. Detillieux K. A. Cattini P. A. (2008). FGF-16 is released from neonatal cardiac myocytes and alters growth-related signaling: a possible role in postnatal development. Am. J. Physiol. Cell Physiol. 294, C1242–C1249 10.1152/ajpcell.00529.2007 18337564 PMC5224940

[B19] Matsumoto E. Sasaki S. Kinoshita H. Kito T. Ohta H. Konishi M. (2013). Angiotensin II-induced cardiac hypertrophy and fibrosis are promoted in mice lacking Fgf16. Genes Cells 18, 544–553 10.1111/gtc.12055 23600527 PMC3738920

[B20] Nickel W. (2010). Pathways of unconventional protein secretion. Curr. Opin. Biotechnol. 21, 621–626 10.1016/j.copbio.2010.06.004 20637599

[B21] Pellieux C. Foletti A. Peduto G. Aubert J. F. Nussberger J. Beermann F. (2001). Dilated cardiomyopathy and impaired cardiac hypertrophic response to angiotensin II in mice lacking FGF-2. J. Clin. Invest. 108, 1843–1851 10.1172/JCI13627 11748268 PMC209469

[B22] Planavila A. Redondo I. Hondares E. Vinciguerra M. Munts C. Iglesias R. (2013). Fibroblast growth factor 21 protects against cardiac hypertrophy in mice. Nat. Commun. 4:2019 10.1038/ncomms3019 23771152

[B23] Rosenkranz S. (2004). TGF-beta1 and angiotensin networking in cardiac remodeling. Cardiovasc. Res. 63, 423–432 10.1016/j.cardiores.2004.04.030 15276467

[B24] Shalhoub V. Shatzen E. M. Ward S. C. Davis J. Stevens J. Bi V. (2012). FGF23 neutralization improves chronic kidney disease-associated hyperparathyroidism yet increases mortality. J. Clin. Invest. 122, 2543–2553 10.1172/JCI61405 22728934 PMC3386816

[B25] Suzuki M. Uehara Y. Motomura-Matsuzaka K. Oki J. Koyama Y. Kimura M. (2008). betaKlotho is required for fibroblast growth factor (FGF) 21 signaling through FGF receptor (FGFR) 1c and FGFR3c. Mol. Endocrinol. 22, 1006–1014 10.1210/me.2007-0313 18187602 PMC5419549

[B26] Touchberry C. D. Green T. M. Tchikrizov V. Mannix J. E. Mao T. F. Carney B. W. (2013). FGF23 is a novel regulator of intracellular calcium and cardiac contractility in addition to cardiac hypertrophy. Am. J. Physiol. Endocrinol. Metab. 304, E863–873 10.1152/ajpendo.00596.2012 23443925 PMC3625783

[B27] Virag J. A. Rolle M. L. Reece J. Hardouin J. S. Feigl E. O. Murry C. E. (2007). Fibroblast growth factor-2 regulates myocardial infarct repair: effects on cell proliferation, scar contraction, and ventricular function. Am. J. Pathol. 171, 1431–1440 10.2353/ajpath.2007.070003 17872976 PMC2043505

[B28] Zhang X. Ibrahimi O. A. Olsen S. K. Umemori H. Mohammadi M. Ornitz D. M. (2006). Receptor specificity of the fibroblast growth factor family. The complete mammalian FGF family. J. Biol. Chem. 281, 15694–15700 10.1074/jbc.M601252200 16597617 PMC2080618

